# Advances in Donkey and Mule Research

**DOI:** 10.3390/ani14152238

**Published:** 2024-08-01

**Authors:** Ana Martins-Bessa, Amy K. McLean

**Affiliations:** 1 Animal and Veterinary Research Centre (CECAV), University of Trás-os-Montes and Alto Douro (UTAD), 5000-801 Vila Real, Portugal; 2 Associate Laboratory for Animal and Veterinary Sciences (AL4AnimalS), 5000-801 Vila Real, Portugal; 3 Department of Animal Sciences, University of California, Davis, CA 95616, USA; acmclean@ucdavis.edu

Donkeys (*Equus asinus*) and mules represent approximately 50% of the entire domestic equine population in the world and play an essential role in the lives of thousands of people, primarily in developing countries [1]. Many donkey breeds are considered endangered and presently facing major problems such as inbreeding, poor reproductive management and old age. On the other hand, there is an increased use for donkeys as pets and production animals and mules as performance animals with superior genetics from both the donkey and horse side, which has increased the interest and value in these animals. New findings focused on improved understanding of basic physiology, behaviour, pain, internal medicine, pathogen frequency, and subjects related to their overall wellbeing (e.g., nutrition, pharmacokinetics, dentistry) are considered areas of interest for preserving and maintaining donkey and mule populations.

Assisted reproductive techniques (ART) like artificial insemination (AI), sperm cryopreservation or embryo transfer (ET) are unique tools to preserve genetic resources. Several studies of the Special Issue were conducted in donkey ART. The study of Lago-Alvarez et al. [2] analyzed the processes of cooling and freezing of epididymal donkey sperm harvested at two different moments: immediately after castration, and 24 or 48 h after shipment. The study allowed the conclusion that freshly harvest and cooled-shipped allowed satisfactory preservation of semen parameters.

Several works were devoted to male donkey reproduction physiology. Martins-Bessa et al. [3] evaluated two predictive models for donkey sperm quality by using the following independent variables: ejaculate’s volume, sperm concentration, total sperm number, motility and sperm morphology. Models included combinations of age as a covariate and biometric and testicular measurements as independent factors. Results evidenced that the goodness-of-fit was similar for both models—hence, the combination of biometry and testicular factors presented improved predictive power. The application of the present models may be useful to gather relevant information that could be used hereafter for ART in donkeys.

Donkeys and mules are treated like horses in many cases, with the physiological differences between these species usually not considered [1]. One significant difference between horses and donkeys’ breeding behaviour is in the additional time donkeys need to achieve erection and ejaculation. Donkeys are characterized by long courtships needed to attain excitation and erection, which can cause donkey semen collections sessions of up to 90 min. The study of Panzani et al. [4] verified the possibility of using Prostaglandin F2α analog, cloprostenol sodium, to hasten the onset of erection and ejaculation. Cloprostenol sodium significantly hastened treatment-to-erection and treatment-to-ejaculation times from 12.0 ± 1.6 to 6.0 ± 1.6 min and from 14.0 ± 1.4 to 9.6 ± 1.4 min, respectively. There were no effects of its administration on semen parameters, being suggested that cloprostenol sodium administration immediately prior to semen collection hastened time to collect semen in donkeys with no detrimental effects on semen quality and can be used by practitioners to circumvent long delays in donkey semen collection. 

The analysis and processing of donkey’s semen for assisted reproductive technologies was also the aim of several works in the Special Issue. The use of CASA (Computer Assisted Sperm Analysis) image analysis system provides an automatic, objective and reliable sperm evaluation; however systematic preparations of the samples should be made. The work of Gacem et al. [5] showed that analyzing a minimum of nine fields at 250 frames per second from the center to the edges in Spermtrack^®^10 chamber using a dilution of 30 × 10^6^ sperm/mL offers the best choice for donkey computerized sperm motility analysis. Semen cryopreservation is also a very important tool for establishing germ plasm banks in order to preserve samples of endangered breeds. In the work of Hidalgo et al. [6], it was found that donkey sperm vitrification in spheres using non-permeable cryoprotectants exhibited better sperm motility and viability parameters after warming than sperm vitrification using extenders containing permeable cryoprotectants. 

The use of single layer centrifugation (SLC) of fresh donkey semen was also investigated and its impact on sperm quality parameters and on the modulation of endometrial reaction following semen deposition using an in vitro model. It was concluded that SLC increases the proportions of functionally intact and motile spermatozoa and appears to remove the seminal plasma proteins that inhibit sperm-PMN binding [7]. 

Also, embryo technology was investigated in donkeys. The work of Dorado et al. [8] aimed to investigate the factors influencing the success of this ART. Donor jenny, donor age, successive cycle within donor, day of flushing, number of flushing, and jack affected embryo recovery rate. The identification of these key points is crucial to achieve a higher efficiency of embryo transfer and vitrification processes, before considering their application in the conservation of endangered donkey breeds.

Research was also conducted in order to find molecular markers that can be used to identify and isolate specific development stages of germ and Leydig cells in the donkey testicle. Several experiments were conducted by Choi et al. [9] with this aim, suggesting that the protein gene product (PGP) 9.5 can be used to identify and isolate spermatogonia at the seminiferous tubules. 

Other articles focused on infectious diseases affecting both donkeys and mules. Realizing that many diseases could compromise donkey well-being, performance and even put the breed at risk in some parts of the world [1]. Review article of these authors provided an update on viruses that may affect donkeys and mules may endanger another species’ health. Health concerns have been raised with the removal and relocation of feral donkeys to offsite adoption locations, as suggested by the work of Goodrich et al. [10]. Previous exposure of the animals to equine herpesvirus 1 (EHV-1), equine influenza (EIV), West Nile virus (WNV), and *Borrelia burgdorferi* (the causative agent of Lyme disease) was assessed. Results indicated that feral equid populations are mostly naïve and likely susceptible to these common equid pathogens upon removal from the wild. Relocation of feral donkeys and possible disease transmission between equine species and the ability to identify early stages of disease by using inflammatory markers such as serum amyloid A (SAA) the aim of the study of Jerele et al. [11]. Results suggest that donkeys do not appear to be a substantial risk for disease transmission to horses but could be if they carried strangles or other processes in which AHV-2 and *Streptococcus zooepidemicus* were involved. The study showed SAA levels may suggest disease as levels were highest in foals who had higher evidence of AHV strains compared to adults. 

The study of Tirosh-Levy et al. [12] evaluated the serologic exposure of donkeys to the parasites *Toxoplasma gondii* and *Neospora* spp. in Israel. The high prevalence found in the study suggested that donkeys may have a role in the maintenance of these parasites in the area, thus serving as a source of infection for the definitive hosts. 

Although belonging to the *Equus* genus, anatomical differences exist, with implications at management and welfare level [13]. This work revealed clear differences at skull morphology between horses and donkeys. The calculation of ratios of skull measurements from horses and donkeys allowed the observation that donkeys skulls have larger forehead than horses and the olfactory bulb was smaller and rotated more forward than horses. 

On the other hand, pharmacological differences between mules and horses were confirmed in the work of Bazzano et al. [14], in which the efficacy and pharmacokinetics of the anthelmintic compound ivermectin administrated orally to mules at the same dosage of horses was evaluated. Results evidenced intermediate pharmacokinetic parameters between horses and donkeys, suggesting an optimization of the dosage. 

Skin diseases caused by fungi and bacteria are quite common in donkeys and mules, which have been reported in several regions of the world as case reports with occasionally retrospective studies. Clinical and pathological features of skin diseases were reviewed and updated by Lima et al. [15], suggesting that geographical variations may results in significant differences in the prevalence of skin diseases. The evaluation of clinical and pathological behaviour of skin diseases directs the implementation of control strategies. 

On the contrary to horses, donkeys tend to express pain and discomfort more subtlety. In consequence, a state of pain could only be recognized in an advanced degree of disease [16]. The study of these authors focused on the development of a scoring system donkey grimace scale based on body language of face and overall body posture and appearance. This system proved to be accurate and effective in identifying discomfort related to pain associated to surgical castration. 

Welfare was also a relevant topic in the Special Issue. Concerning reports of welfare issues were related in the work of Farhat et al. [17], with working donkeys in Egypt showing many types of wounds associated with parts of the harness and use of excessive force or beating. The study introduced methods to measure the welfare status in working donkeys and found an association between health risks, behavioral parameters and body condition. Overall welfare of working donkeys should be enhanced in order to improve the production of the animals and the reduction of the wounds. Besides, observation of the body condition should be promoted together with the owners, in order to avoid compromising the body condition and welfare. 

In countries like France, Italy and Brazil the asinine milk production has some economic relevance due to the milk qualities for human consumption. The use of donkeys for this purpose must guarantee their welfare [18]. The work of these researchers investigated whether a 2h separation period, used to achieve milking efficiency, was stressful for jennies and their foals. For that, the behavioural and physiological changes of the animals when exposed to the milking routine were assessed. Behavioral assessments and determination of cortisol salivary concentration were collected before and after separation in jennies and foals. Considering the collected variables, results of the work suggest that the milking routine did not appear to be stressful for the animals. More studies are desirable in order to measure the impact of milking routine and to guarantee the welfare of the animals in that industry. 

The role of donkeys in the reduction of fuel biomass of forest was verified in the work of Bartolomé at al. [19], being suggested the combined use of donkeys and goats, as both animals feed on forest species, and could act complementary to prevent fire in the Mediterranean forests. 

Some works of the Special Issue improved the knowledge about medical conditions in donkeys, which will contribute to the provision of appropriate medical care. The asinine metabolic syndrome and the pituitary pars intermedia dysfunction were reviewed by Gehlen et al. [20]. A special emphasis was given in this review to the differences evidenced in donkeys, as reference values and physiological reaction to dynamic tests. 

Some health issues are still neglected in donkeys. Dental health has been neglected in donkeys [21], despite the frequency of dental problems in this specie. The work of these authors contributed to knowledge of donkey oral microbiome sequencing in association with dental care treatment. Future works should promote a more abroad understanding of the impact of disease as periodontitis in donkey dental health [21]. 

The study of passive transfer of immunity in donkey foals compared to horse foals has been studied very little. The work of Turini et al. [22] determined the IgG serum concentration in donkeys’ foals by different analytic methods in the first 24 h. Methods revealed good or strong correlation to each other. Based on feasibility, serum total protein (TP) refractometry could be a useful method to estimate transfer of immunity to donkey foals. 

The infrared thermography was applied comparatively in donkeys and horses in the work of Domino et al. [23]. Similarities were found in thermal patterns of both species; however average surface temperatures were higher in horses which was related to different thermal properties of the skin and hair coat.

Finally, two case reports described the implantation of a transvenous single-chamber pacemaker due to severe bradycardic arrythmia in five-month-old jenny with successful outcome [24] and the medical management of four donkeys with various degrees of external severe burn injuries along with clinical findings and evolution [25]. 
